# Supplementary material for: Limits to detecting epistasis in the fitness landscape of HIV
Source: PLoS One. 2022 Jan 18;17(1):e0262314. doi: 10.1371/journal.pone.0262314 (PMC8765623; doi:10.1371/journal.pone.0262314)
Supplement: S1 File — Details of the evolutionary conservation in different HIV enzymatic proteins and its effect on the observable evidence for epistasis is given in Section 1. Details on comparison with different experimental measures of fitness and Potts and independent models, along with comparisons with FoldX predicted changes in folding energetics due to mutations are given in Section 2, as well as details of why comparisons for double mutant cycles are difficult. Details of the weak evidence for epistasis that can be drawn from hamming distance distributions are given in Section 3. (PDF) [file pone.0262314.s001.pdf]

Supplementary File 1
Limits to detecting epistasis in the fitness landscape of HIV

Avik Biswas[1,2], Allan Haldane[1,2], and Ronald M Levy[1,2,3]

[1] *Department of Physics, Temple University, Philadelphia, PA*
[2] *Center for Biophysics and Computational Biology, Temple University, Philadelphia, PA*
[3] *Department of Chemistry, Temple University, Philadelphia, PA*

1 Evolutionary conservation in HIV enzymes and its effect on correlations between sites

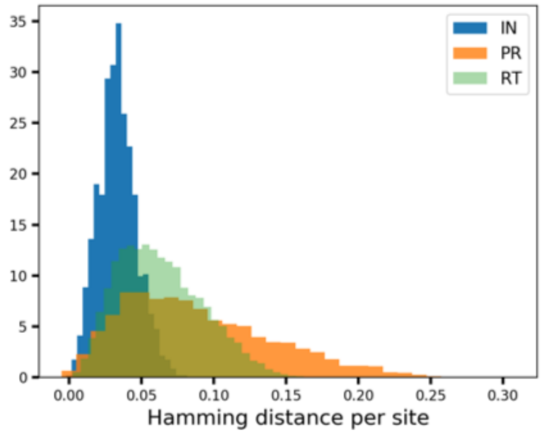

Figure 1: **Degree of conservation in HIV enzymes**. Distribution of the average number of mutations per site from the reference HIV-1 subtype B consensus sequence (Hamming distances) is compared between HIV protease (PR), reverse transcriptase (RT), and integrase (IN) proteins. Protease sequences have much more average variation per site compared to RT and IN, and is the least conserved amongst the *Pol* enzymes of HIV, followed by RT, and then IN, which has similar degree of conservation comparable to structural proteins like p24 capsid (CA).

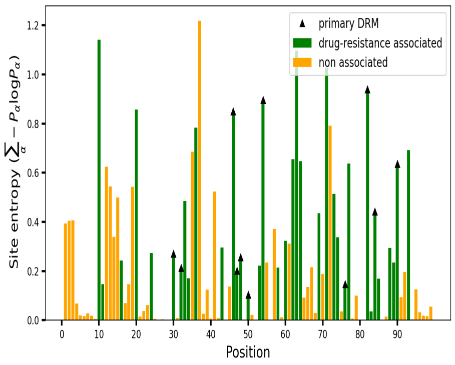

(A) Site entropy in protease.

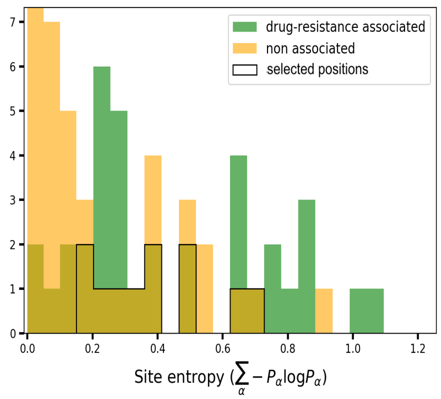

(B) Distribution of site entropies at resistance-associated and non-associated sites.

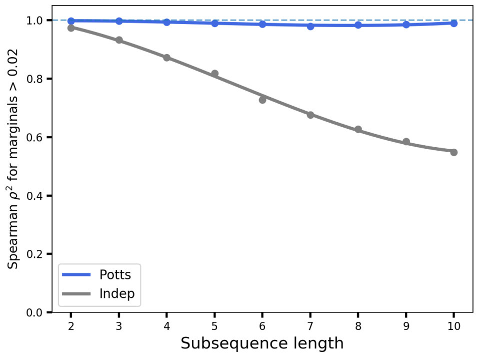

(C) Higher order marginals for selected resistance-associated sites.

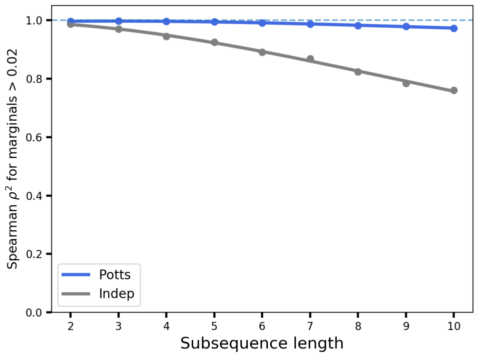

(D) Higher order marginals for selected non-associated sites.

Figure 2: **Lack of variability at residue positions can affect the observed correlations.** (A) shows the site entropies ($\sum_\alpha -P_\alpha log P_\alpha$, where the sum runs over the residues at that position) at drug-resistance associated sites in green and non-drug-resistance associated sites in orange. In the reduced alphabet of 4 as used here, there are 4 possible amino acid characters in each position. Sites of primary mutations are indicated with a black "triangle" symbol. Site entropy is a measure for the variability at a site. (B) shows the distribution of site entropies for drug-resistance associated positions in green, non-drug-resistance associated positions in orange, and the overlap between them in brown. There is higher variability, i.e higher site entropy at positions associated with drug resistance. Both resistance-associated and non-associated sites are then selected for further analysis such that they have similar distribution of site entropies as indicated in black. (Again a reduced alphabet of 4 was used here, giving 4 possible amino-acid characters at each site in the protein.) (C) Spearman ρ^2 between the Potts (blue) or independent (gray) model predictions and dataset marginals for subsequences of length upto 10 comprised of selected inhibitor-associated positions with similar distributions in site entropies as non associated positions shown in (B). (D) Same as (C) but for subsequences comprised of selected positions not associated with resistance. While lack of variability at sites can play a role in obscuring the effect of correlations, the role of correlations is still stronger for inhibitor-associated sites than can accounted for by site entropies alone, and is possibly due to the functional interactions connecting them.

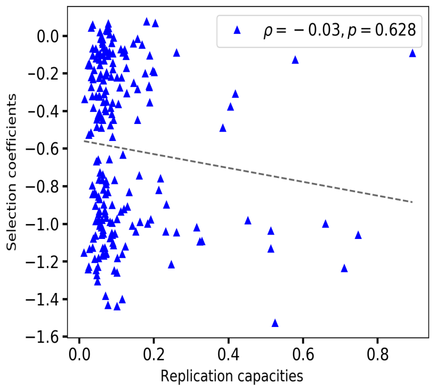

Figure 3: Replicative capacity measurements from [1] compared to selection coefficients from [2] for mutations in HIV-1 Protease.

2 Comparison with experimental measures of fitness

Figure 3 compares two very similar experimental measures of fitness: replicative capacity measurements from [1] with selection coefficients from [2] for mutations in HIV-1 Protease. The correlation between the two is low. Interestingly, the Potts model predictions agree well with one of the datasets and not the other.

Figure 4 illustrates the correspondence between the likelihoods of point mutations in HIV protease (PR) as predicted by the Potts model and three different but related experimental measures of fitness: relative infectivity measured by single-cycle specific infectivity (SpIn) assays with the amount of viruses for infection normalized by measuring p24 mass (ELISA) from [3] in (A), replication capacity values of viruses with single mutations associated with protease inhibitor resistance from [3] in (B), replication capacity measurements from [1] in (C), and selection coefficients from [2] in (D). There is high, statistically significant correlation between Potts model predictions of likelihoods and experimental fitness measurements in (A, B, and D). [4] also report a Pearson's correlation coefficient, $|R| \approx 0.85$ between changes in melting temperature (T_m) of mutant proteases relative to a reference sequence and Potts ΔEs. However, there is low correspondence between the high-throughput replicative capacity index in [1] and predicted likelihoods of single mutations in protease in Figure 4C, which illustrates the fact that the same experimental measurement depending on context, type of experiment, experimental conditions, etc. can have varying degrees of correlation with predicted "likelihoods" of mutant sequences. The model predictions can also be contaminated if the measurements are for mutations rarely observed in large MSAs of natural sequences.

Figure 8 illustrates an interpretation that the Potts model predicted ΔEs are representative of a collective of different features in the fitness landscape, such as viral replicative capacities, or protein structural stabilities (and folding energies), which may be orthogonal, and thus, may not correlate well with each other. Potts model predicted ΔEs correlate well with both replicative capacities of protease mutants, as well as with changes in their folding energies predicted by FoldX. Replicative capacities however do not correlate as well with FoldX predicted changes in folding energies. Using an adaptive cluster expansion algorithm to infer the Potts model parameters, [6] also report a statistically significant correlation between replicative capacities and Potts model predicted ΔEs for second-generation drug-associated mutations in HIV PR.

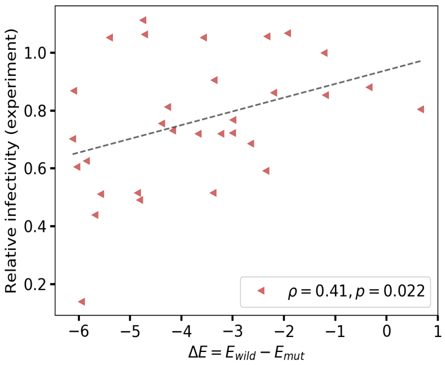

(A) Relative infectivity measured using single cycle SpIn assays [3] vs Potts model predicted likelihoods

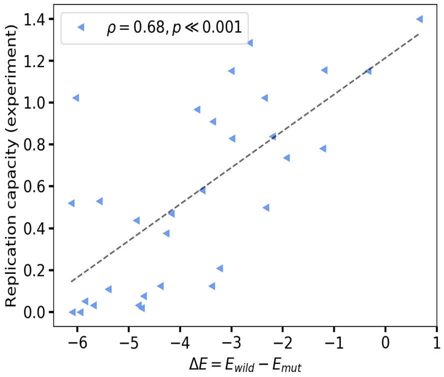

(B) Replication capacity measurements from [3] vs Potts model predicted likelihoods

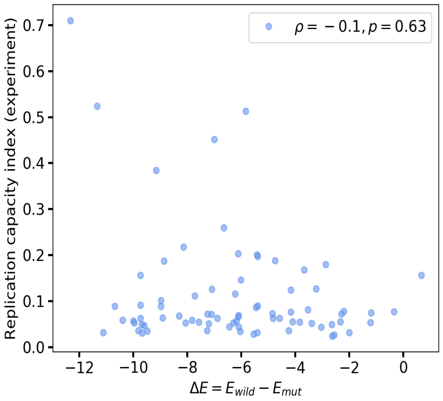

(C) Replication capacity measurements from [1] vs Potts model predicted likelihoods

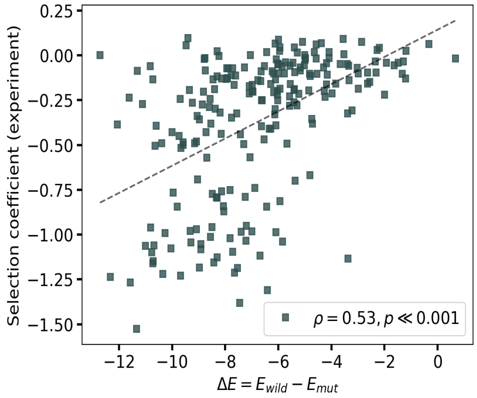

(D) Selection coefficients from [2] vs Potts model predicted likelihoods

Figure 4: **Same sequence covariation model, different experimental measurements**. Figure shows the correlation between the likelihoods of point mutations in the Protease enzyme of HIV-1 as predicted by the same sequence covariation (Potts) model and four different but related experimental measures of fitness: (A) relative infectivity measured using single cycle specific infectivity (SpIn) assays [3] , (B), viral replication capacity measurements from [3], and (C) replication capacity measurements from [1], and selection coefficients from [2] in (D). (A) Changes in relative infectivity measured by single-cycle SpIn assay, in which the amount of viruses used for infection was normalized by measuring p24 mass (ELISA) from [3] compared to Potts ΔEs. Spearman $\rho = 0.41$ (Pearson $R = 0.38$). (B) Replication capacity values of viruses with single mutations associated with protease inhibitor resistance from [3] compared to Potts ΔEs. Spearman $\rho = 0.68$ (Pearson $R = 0.71$). (C) Replication capacity measurements obtained by high density mutagenesis experiments combined with next-generation sequencing in [1] compared to Potts ΔEs. Spearman $|\rho| = 0.1$ (Pearson $|R| = 0.24$). (D) Selection coefficients from [2] compared to Potts ΔEs. Spearman $\rho = 0.53$ (Pearson $R = 0.50$). [4] report a Pearson's correlation coefficient, $|R| \approx 0.85$ between changes in melting temperature (T_m) of mutant HIV proteases relative to a reference sequence and Potts ΔEs.

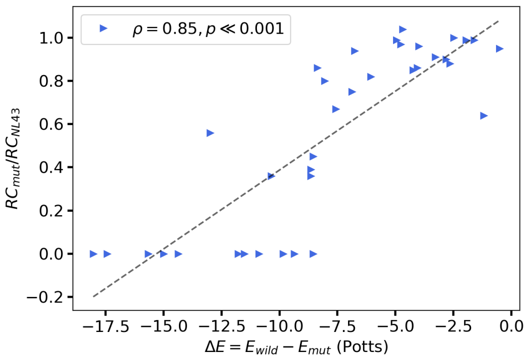

(A) Replicative fitness from [5] vs Potts model predicted likelihoods in p24 capsid.

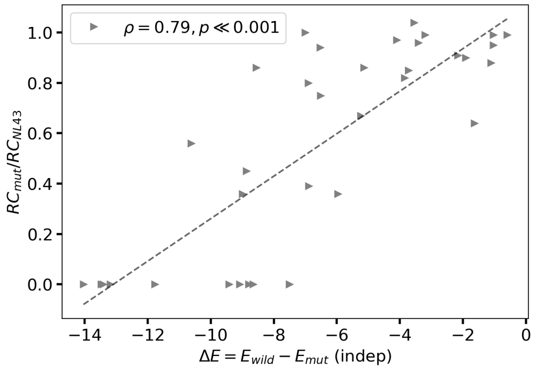

(B) Replicative capacity measurements from [5] vs independent model predicted likelihoods in p24 capsid.

Figure 5: **Experimental measurements of replicative capacity vs model predicted likelihoods of mutations in HIV capsid protein**. Figure shows the correlation between the likelihoods of point mutations in p24 capsid as predicted by the Potts and independent models compared to experimental measures of replicative fitness (RC_{mut}/RC_{NL43}) from [5], with a Spearman rank-order correlation coefficient of 0.85 and 0.79, respectively.

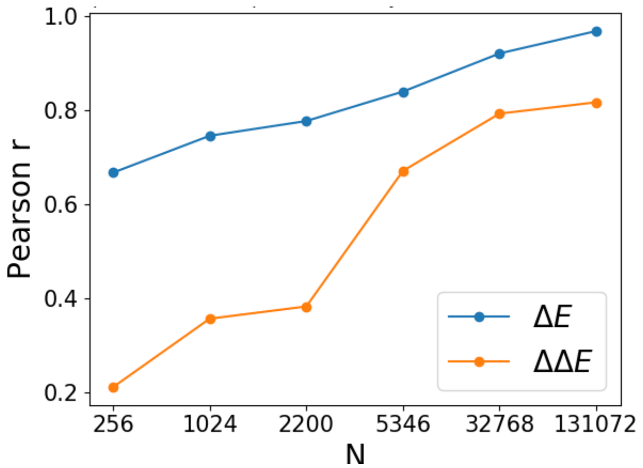

Figure 6: (A) Accuracy of point-mutation effect predictions and "double mutant cycle" predictions of epistasis for HIV-1 capsid, as a function of the MSA depth from which the Potts model was inferred. This is measured by the Pearson correlation in mutation effect predictions ΔE for all possible mutations and $\Delta\Delta E$ for all possible double mutants to a set of sequences generated from the reference model.

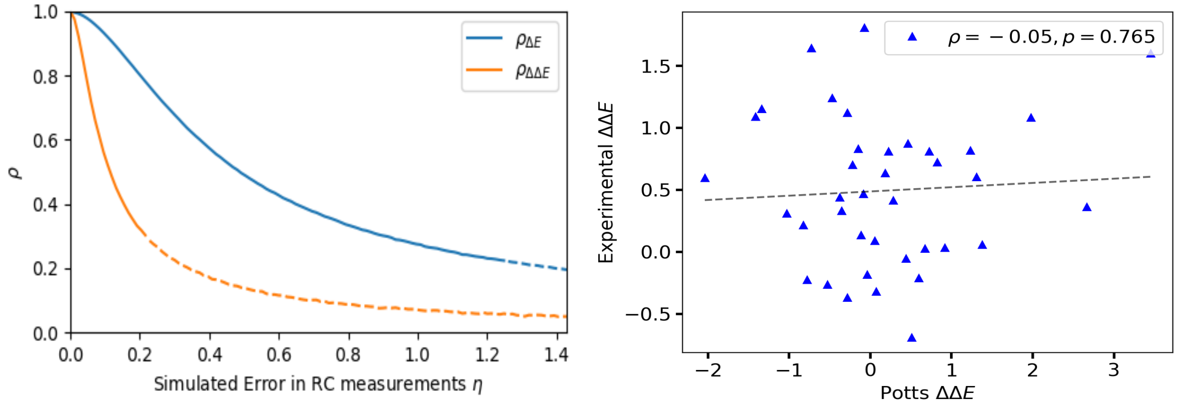

(A) (B)

Figure 7: (A) Simulation of the expected correlation of the Potts model prediction to experimental values for ΔE and $\Delta\Delta E$ in HIV-1 protease as a function of simulated experimental noise η, showing that the the correlation for $\Delta\Delta E$ drops much more quickly. The dotted section of the curves show where the p-value for the $\Delta\Delta E$ correlation is > 0.05, or insignificant, showing that noise can make it impossible to verify $\Delta\Delta E$ values even when ΔE values are well predicted. For a correlation of $\rho \approx 0.6$ for ΔE values between model and experiment (as seen in main text Figure 4 for protease), the expected correlation in $\Delta\Delta E$ values is ≈ 0.2 and is not statistically significant. (B) Comparison of double mutant cycle predictions between Potts model and experimental measurements of relative replicative fitness from [6] for HIV-1 PR. The much smaller dynamic range makes the measurements/predictions more susceptible to noise, making accurate numerical predictions difficult. We do not see statistically significant numerical correlation between experimental and Potts $\Delta\Delta E$ predictions.

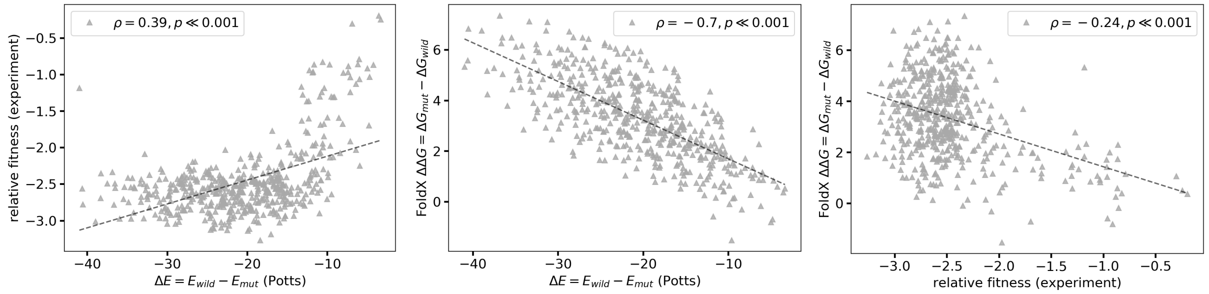

(A) (B) (C)

Figure 8: **Potts model captures different features of the fitness landscape**. Figure shows that the Potts model predicted ΔEs can capture different features of the fitness landscape that may be orthogonal, and may not correlate well with each other. (A) Relative fitness (replicative capacity) measurements obtained from deep mutational scanning of HIV-1 variants [6] involving combinations of (upto 8) mutations in protease associated with resistance to (particularly second-generation) inhibitors in clinic, are compared to changes in Potts statistical energies, ΔEs with a Spearman rank-order correlation, $\rho \approx 0.4$ ($p \ll 0.001$). The observed correlation between model and experiment is lower than seen in the main text as the experimental dataset contains combinations of mutations not observed in patients, which are highly deleterious (particularly for higher order mutants) and may suffer from higher experimental errors [6]. Excluding higher-order mutants and very deleterious genotypes was also seen to improve the correlation between the Potts model predictions and experimental fitness measurements in [6] (B) FoldX predicted changes in folding energies, $\Delta\Delta G$s (PDB: 3S85) of the mutations also correlate well with Potts predicted changes in statistical energies, ΔEs for the same (Spearman $\rho \approx -0.7$). (C) Experimental relative fitness measurements however, do not correlate as well with FoldX predicted changes in folding energies due to the mutations ($\rho = -0.24$).

3 Evidence of epistasis from hamming distance distributions

In this section, we look at the evidence of epistasis that can be obtained from the distinction in the hamming distance (number of mutations) distributions of the Potts and independent models compared to the distribution in the multiple sequence alignment of protein sequences (dataset MSA) obtained from the Stanford HIV drug resistance database, using total variation distance (TVD) as a measure of the distance between distributions. Samples (of the same size as the dataset MSA) are obtained by sampling from the Potts and independent models, as well as the dataset MSA itself, giving the "target" distribution. Hamming distances are calculated with respect to the HIV-1 subtype B wild-type consensus sequence. The total variation distances of the hamming distance (inclusive of all positions) distributions are calculated with respect to the dataset MSA, and the process is repeated to obtain the distribution of total variation distances (Figure 9). The Potts model predicted hamming distance distributions are very close to the "target" distribution. The biggest difference between the Potts and independent models is seen for drug-experienced protease in Figure 9B. However, in most of the literature, drug-naive HIV sequences are used, where the distinction between Potts and independent models is not clear (Figure 9A). For integrase, which is a much more conserved protein than protease, the models are indistinguishable even for drug-experienced sequences (Figure 9C). Overall, the evidence of epistasis that can be obtained from hamming distance distributions is not strong and less sensitive than can be obtained from higher-order mutational statistics.

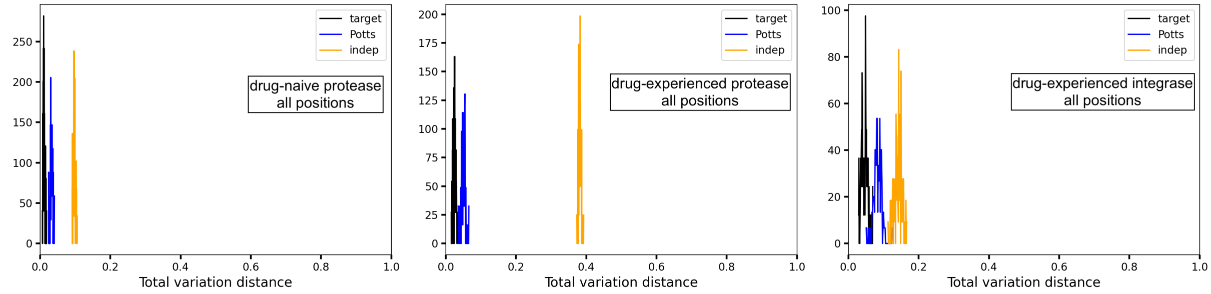

(A) (B) (C)

Figure 9: **Distribution of the total variation distances of hamming distance distributions**. Figure shows the distribution of the total variation distances in the Hamming distances (from the HIV-1 subtype B consensus sequence considering all positions in the protein) of the Potts model (blue), independent model (orange) and target (black) distributions, of which the latter is obtained by repeated sampling from the dataset MSA. The total variation distance of each distribution is calculated with respect to the dataset MSA and shown for (A) drug-experienced protease, (B) drug-naive protease, and (C) drug-experienced integrase. The biggest distinction between the Potts and independent models is seen for drug-experienced protease. However, in most of the literature, drug-naive HIV sequences are used, where the distinction between Potts and independent models is not clear. For integrase, which is a much more conserved protein than protease, the models are almost indistinguishable even for drug-experienced sequences. Overall, the evidence of epistasis that can be obtained from hamming distance distributions is not strong and less sensitive than that obtained from higher-order mutational statistics.

References

[1] Laith Q Al-Mawsawi, Nicholas C Wu, C Anders Olson, Vivian Cai Shi, Hangfei Qi, Xiaojuan Zheng, Ting-Ting Wu, and Ren Sun. High-throughput profiling of point mutations across the hiv-1 genome. *Retrovirology*, 11(1):124, 2014.

[2] Jeffrey I Boucher, Troy W Whitfield, Ann Dauphin, Gily Nauchum, Konstantin B Zeldovich, Ronald Swanstrom, Celia A Schiffer, Jeremy Luban, and Daniel NA Bolon. Constrained mutational sampling of amino acids in hiv-1 protease evolution. *bioRxiv*, page 354597, 2019.

[3] Gavin J Henderson, Sook-Kyung Lee, David M Irlbeck, Janera Harris, Melissa Kline, Elizabeth Pollom, Neil Parkin, and Ronald Swanstrom. Interplay between single resistance-associated mutations in the hiv-1 protease and viral infectivity, protease activity, and inhibitor sensitivity. *Antimicrobial agents and chemotherapy*, 56(2):623–633, 2012.

[4] William F Flynn, Allan Haldane, Bruce E Torbett, and Ronald M Levy. Inference of epistatic effects leading to entrenchment and drug resistance in hiv-1 protease. *Molecular biology and evolution*, 34(6):1291–1306, 2017.

[5] Jaclyn K Mann, John P Barton, Andrew L Ferguson, Saleha Omarjee, Bruce D Walker, Arup Chakraborty, and Thumbi Ndung'u. The fitness landscape of hiv-1 gag: advanced modeling approaches and validation of model predictions by in vitro testing. *PLoS computational biology*, 10(8):e1003776, 2014.

[6] Tian-hao Zhang, Lei Dai, John P Barton, Yushen Du, Yuxiang Tan, Wenwen Pang, Arup K Chakraborty, James O Lloyd-Smith, and Ren Sun. Predominance of positive epistasis among drug resistance-associated mutations in hiv-1 protease. *PLoS genetics*, 16(10):e1009009, 2020.
