# Supplementary material for: Limits to detecting epistasis in the fitness landscape of HIV
Source: PLoS One. 2022 Jan 18;17(1):e0262314. doi: 10.1371/journal.pone.0262314 (PMC8765623; doi:10.1371/journal.pone.0262314)
Supplement: S2 File — Figures and tables showing the distribution of Potts model predicted double mutant cycle effects for all double mutations indicating the strongest, predicted double mutant cycle effects involving mutations (at least one amongst the pair) at drug-resistance-associated sites and corresponding literature references in HIV-1 protease and integrase are given. (PDF) [file pone.0262314.s002.pdf]

Supplementary File 2

Limits to detecting epistasis in the fitness landscape of HIV

Avik Biswas[1,2], Allan Haldane[1,2], Ronald M Levy[1,2,3]

[1]Department of Physics, Temple University, Philadelphia, PA
[2]Center for Biophysics and Computational Biology, Temple University, Philadelphia, PA
[3]Department of Chemistry, Temple University, Philadelphia, PA

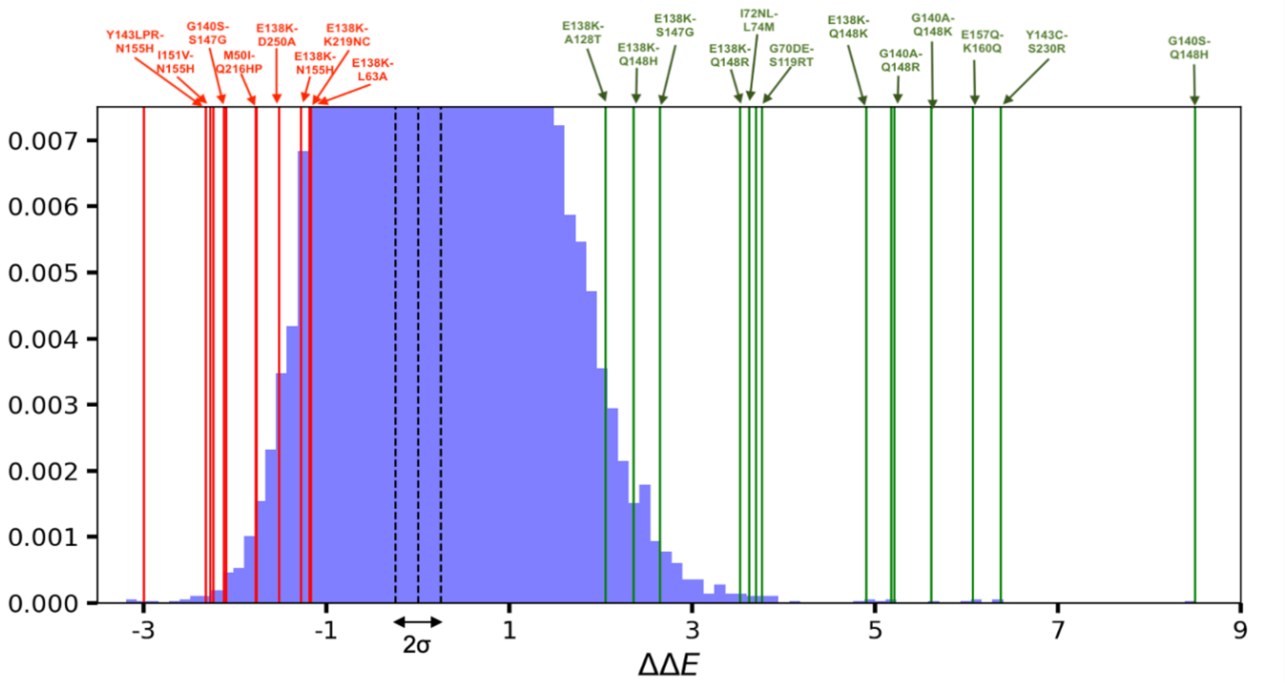

Figure S2A: *Figure shows the distribution of Potts model predicted double mutant cycles ($\Delta\Delta E$) in HIV-1 Integrase (blue) with respect to the NL4-3 background. $\Delta\Delta E$ values above zero (0) indicate positive epistasis while $\Delta\Delta E$ values below zero (0) indicate negative epistasis. The location of zero (0) and the standard deviations of $\Delta\Delta E$ are shown as black 'dashed' lines. Some of the strongly interacting double mutations which involve drug-resistance mutations are indicated and labelled by colored lines; and are studied in the literature. Many of the strongest double mutant cycles captured by the Potts model are for drug-resistance mutations.*

<u>Supplementary Table S2A:</u> Potts model predicted strongest, positive double mutant cycle effects involving mutations (at least one amongst the pair) at drug-resistance-associated sites with corresponding literature references in HIV-1 Integrase.

**Mutation pair**	Potts $\Delta\Delta E$	**Literature reference***
G140S-Q148H	8.50879	1, 2, 3, 4, 5
Y143C-S230R	6.37754	6
E157Q-K160Q	6.0882	---
G140A-Q148K	5.61299	1, 2, 3, 4
G140A-Q148R	5.2042	1, 2, 3, 4
G140A-Q148K	4.91495	1, 2, 3, 4
E138K-Q148K	4.90052	5, 6, 7, 8
G70DE-S119RT	3.76054	----
Y143ASGH - S230R	3.7007	6
I72NL-L74M	3.62676	9
E138K-Q148R	3.5258	4, 6, 7, 8
E138K-Q148H	2.3615	4,6,7,8

* Literature references are mentioned only for pairs where the coupled effect of the mutations is studied in literature.

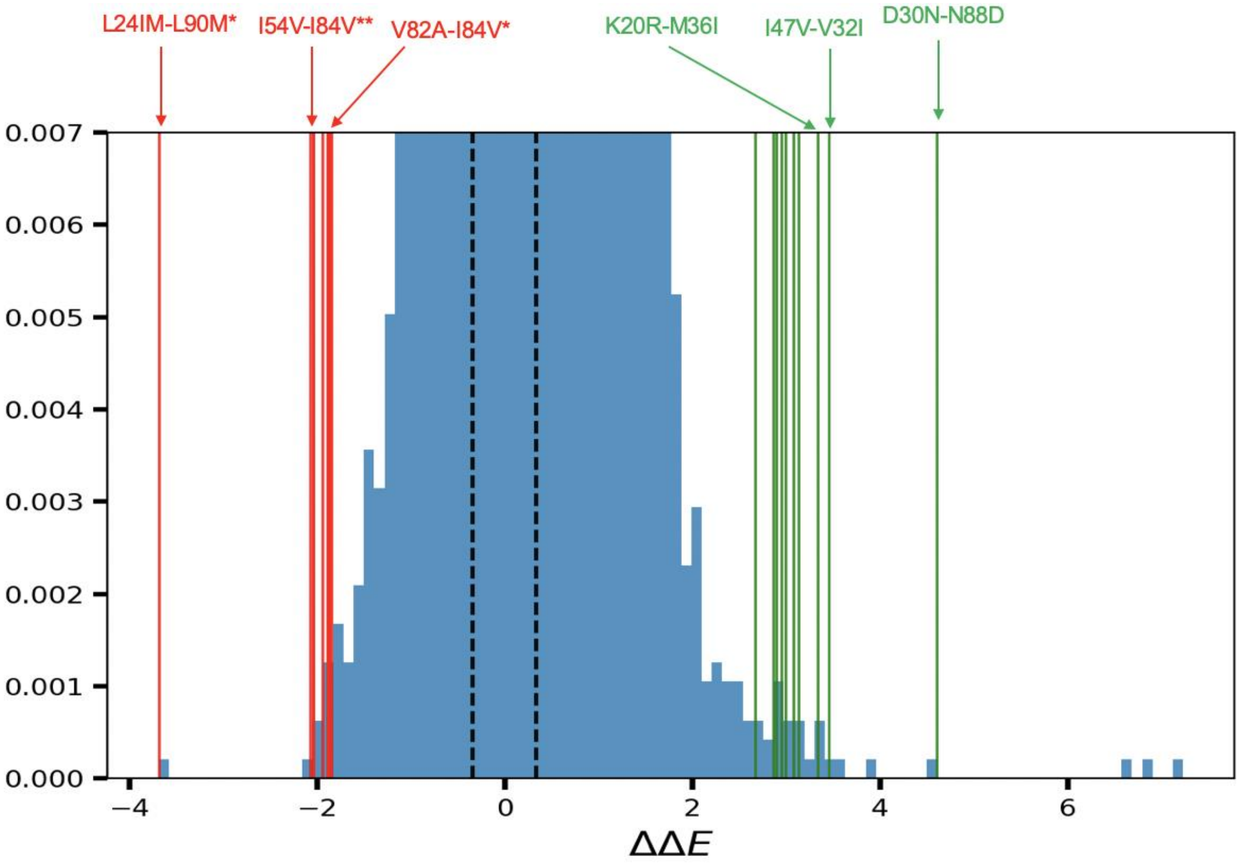

Figure S2B: *Figure shows the distribution of Potts model predicted double mutant cycles (ΔΔE) in HIV-1 Protease (blue). ΔΔE values above zero (0) indicate positive epistasis while ΔΔE values below zero (0) indicate negative epistasis. The location of zero (0) and the standard deviations of ΔΔE are shown as black 'dashed' lines. Some of the strongly interacting double mutations which involve drug-resistance mutations are indicated and labelled by colored lines; and are studied in the literature. Many of the strongest double mutant cycles captured by the Potts model are for drug-resistance.*

<u>Supplementary Table S2B:</u> Potts model predicted strongest, positive double mutant cycle effects involving mutations (at least one amongst the pair) at drug-resistance-associated sites with corresponding literature references in HIV-1 Protease.

Mutation pair	$Potts\ \Delta\Delta E$	Literature reference*
D30N-N88D	4.61	10, 11, 13, 14
I47V—V32I	3.45	15, 16
K20R-M36I	3.33	17
G48VALMQ-I54ATS	3.14	---
D30N-K45Q	3.08	----
G16E-P39S	2.99	---
I54ATS-V82A	2.95	12, 13, 18
P39S-I62K	2.89	----
I54V-V82A	2.86	21
M46I-L76V	2.67	14, 19, 20

* Literature references are mentioned only for pairs where the coupled effect of the mutations is studied in literature.

References:

1. Blanco, J.L., Varghese, V., Rhee, S.Y., Gatell, J.M. and Shafer, R.W., 2011. HIV-1 integrase inhibitor resistance and its clinical implications. *Journal of Infectious Diseases*, *203*(9), pp.1204-1214.
2. Molina, J.M., LaMarca, A., Andrade-Villanueva, J., Clotet, B., Clumeck, N., Liu, Y.P., Zhong, L., Margot, N., Cheng, A.K. and Chuck, S.L., 2012. Efficacy and safety of once daily elvitegravir versus twice daily raltegravir in treatment-experienced patients with HIV-1 receiving a ritonavir-boosted protease inhibitor: randomised, double-blind, phase 3, non-inferiority study. *The Lancet infectious diseases*, *12*(1), pp.27-35.
3. Garrido, C., Villacian, J., Zahonero, N., Pattery, T., Garcia, F., Gutierrez, F., Caballero, E., Van Houtte, M., Soriano, V. and de Mendoza, C., 2012. Broad phenotypic cross-resistance to elvitegravir in HIV-infected patients failing on raltegravir-containing regimens. *Antimicrobial agents and chemotherapy*, *56*(6), pp.2873-2878.
4. Fransen, S., Gupta, S., Frantzell, A., Petropoulos, C.J. and Huang, W., 2012. Substitutions at amino acid positions 143, 148, and 155 of HIV-1 integrase define distinct genetic barriers to raltegravir resistance in vivo. *Journal of virology*, *86*(13), pp.7249-7255.
5. Cook, N.J., Li, W., Berta, D., Badaoui, M., Ballandras-Colas, A., Nans, A., Kotecha, A., Rosta, E., Engelman, A.N. and Cherepanov, P., 2020. Structural basis of second-generation HIV integrase inhibitor action and viral resistance. *Science*, *367*(6479), pp.806-810.
6. Eron, J.J., Clotet, B., Durant, J., Katlama, C., Kumar, P., Lazzarin, A., Poizot-Martin, I., Richmond, G., Soriano, V., Ait-Khaled, M. and Fujiwara, T., 2013. Safety and efficacy of dolutegravir in treatment-experienced subjects with raltegravir-resistant HIV type 1 infection: 24-week results of the VIKING Study. *The Journal of infectious diseases*, *207*(5), pp.740-748.
7. Canducci, F., Ceresola, E.R., Boeri, E., Spagnuolo, V., Cossarini, F., Castagna, A., Lazzarin, A. and Clementi, M., 2011. Cross-resistance profile of the novel integrase inhibitor Dolutegravir (S/GSK1349572) using clonal viral variants selected in patients failing raltegravir. *Journal of Infectious Diseases*, *204*(11), pp.1811-1815.
8. Cahn, P., Pozniak, A.L., Mingrone, H., Shuldyakov, A., Brites, C., Andrade-Villanueva, J.F., Richmond, G., Buendia, C.B., Fourie, J., Ramgopal, M. and Hagins, D., 2013. Dolutegravir versus raltegravir in antiretroviral-experienced, integrase-inhibitor-naive adults with HIV: week 48 results from the randomised, double-blind, non-inferiority SAILING study. *The Lancet*, *382*(9893), pp.700-708.
9. Kobayashi, M., Nakahara, K., Seki, T., Miki, S., Kawauchi, S., Suyama, A., Wakasa-Morimoto, C., Kodama, M., Endoh, T., Oosugi, E. and Matsushita, Y., 2008. Selection of diverse and clinically relevant integrase inhibitor-resistant human immunodeficiency virus type 1 mutants. *Antiviral research*, *80*(2), pp.213-222.
10. Atkinson, B., Isaacson, J., Knowles, M., Mazabel, E. and Patick, A.K., 2000. Correlation between human immunodeficiency virus genotypic resistance and virologic response in patients receiving nelfinavir monotherapy or nelfinavir with lamivudine and zidovudine. *The Journal of infectious diseases*, *182*(2), pp.420-427.
11. Patick, A.K., Duran, M., Cao, Y., Shugarts, D., Keller, M.R., Mazabel, E., Knowles, M., Chapman, S., Kuritzkes, D.R. and Markowitz, M., 1998. Genotypic and phenotypic characterization of human immunodeficiency virus type 1 variants isolated from patients treated with the protease inhibitor nelfinavir. *Antimicrobial Agents and Chemotherapy*, *42*(10), pp.2637-2644.
12. Podzamczer, D., Ferrer, E., Consiglio, E., Gatell, J.M., Perez, P., Perez, J.L., Luna, E., González, A., Pedrol, E., Lozano, L. and Ocaña, I., 2002. A randomized clinical trial comparing nelfinavir or nevirapine associated to zidovudine/lamivudine in HIV-infected naive patients (the Combine Study). *Antiviral therapy*, *7*(2), pp.81-90.
13. Fitzgibbon, J.E., Gaur, S, Walsman, S.M., Janahi, M, Whitley-Williams, P, and John, J.F. Jr., 2001. Emergence of drug resistance mutations in a group of HIV-infected children taking nelfinavir-containing regimens. *AIDS Res Hum Retroviruses*, 17(14),1321-1328.
14. Rhee, S.Y., Taylor, J., Fessel, W.J., Kaufman, D., Towner, W., Troia, P., Ruane, P., Hellinger, J., Shirvani, V., Zolopa, A. and Shafer, R.W., 2010. HIV-1 protease mutations and protease inhibitor cross-resistance. *Antimicrobial agents and chemotherapy*, *54*(10), pp.4253-4261.
15. Rhee, S.Y., Liu, T.F., Holmes, S.P. and Shafer, R.W., 2007. HIV-1 subtype B protease and reverse transcriptase amino acid covariation. *PLoS Comput Biol*, *3*(5), p.e87.

16. Kagan, R.M., Shenderovich, M.D., Heseltine, P.N. and Ramnarayan, K., 2005. Structural analysis of an HIV-1 protease I47A mutant resistant to the protease inhibitor lopinavir. *Protein science*, *14*(7), pp.1870-1878.
17. Nijhuis, M., Schuurman, R., De Jong, D., Erickson, J., Gustchina, E., Albert, J., Schipper, P., Gulnik, S. and Boucher, C.A., 1999. Increased fitness of drug resistant HIV-1 protease as a result of acquisition of compensatory mutations during suboptimal therapy. *Aids*, *13*(17), pp.2349-2359.
18. van Westen, G.J., Hendriks, A., Wegner, J.K., IJzerman, A.P., van Vlijmen, H.W. and Bender, A., 2013. Significantly improved HIV inhibitor efficacy prediction employing proteochemometric models generated from antivirogram data. *PLoS Comput Biol*, *9*(2), p.e1002899.
19. Henderson, G.J., Lee, S.K., Irlbeck, D.M., Harris, J., Kline, M., Pollom, E., Parkin, N. and Swanstrom, R., 2012. Interplay between single resistance-associated mutations in the HIV-1 protease and viral infectivity, protease activity, and inhibitor sensitivity. *Antimicrobial agents and chemotherapy*, *56*(2), pp.623-633.
20. Naeger, L.K. and Struble, K.A., 2007. Food and Drug Administration analysis of tipranavir clinical resistance in HIV-1-infected treatment-experienced patients. *Aids*, *21*(2), pp.179-185.
21. Bethell, R., Scherer, J., Witvrouw, M., Paquet, A., Coakley, E. and Hall, D., 2012. Phenotypic protease inhibitor resistance and cross-resistance in the clinic from 2006 to 2008 and mutational prevalences in HIV from patients with discordant tipranavir and darunavir susceptibility phenotypes. *AIDS research and human retroviruses*, *28*(9), pp.1019-1024.
