# Supplementary material for: Limits to detecting epistasis in the fitness landscape of HIV
Source: PLoS One. 2022 Jan 18;17(1):e0262314. doi: 10.1371/journal.pone.0262314 (PMC8765623; doi:10.1371/journal.pone.0262314)
Supplement: Attachment — Submitted filename: PlosOne_response.pdf [file pone.0262314.s003.pdf]

Editors' comments to the author:

Reviewer 1 strongly criticizes the way you present the model but gives you the benefit of the doubt about the correctness of your methodology. Reviewer 2 on the other hand finds your results to be inconclusive and therefore of limited biological significance. Based on these expert assessments we cannot accept your manuscript.

We are disappointed with the editor's decision to not consider the manuscript for publication based on the two reviews. While reviewer 1 criticizes our presentation of the model, he comments on finding the results reported in our manuscript satisfying and of importance; and states that he "has no reason to doubt the authors used the model correctly, nor do I doubt their results." Reviewer 1 asks that we improve our presentation of the model and objects to us referring to the model as a "Potts model". While we agree that the model presented in this manuscript and in the literature cited by the reviewer is not precisely the Potts model from statistical physics, it is a generalization of the same model and is often referred to simply as a "Potts model" in the biological physics literature when applied to protein sequence co-variation. We clarify the use of the name "Potts model" in the revised manuscript, and also address several additional points raised by reviewer 1 in our further response below.

As to the comments from reviewer 2, we strongly disagree that the results are inconclusive and highlight below some of the new results presented in the manuscript that will be of importance to the understanding and detection of epistasis in viral systems more generally and for HIV in particular.

Our work is motivated by recent publications [1] which show that when experimental probes of epistasis, primarily based on replicative capacity, are compared with <u>global</u> models of sequence co-variation for HIV proteins, the signatures of epistasis are weak for these viral proteins. As discussed in our manuscript and not well understood by the scientific community, HIV replicative capacity assays are explained surprisingly well using an independent model of sequence evolution. Yet, the role played by epistasis in the evolution of viruses is correctly thought to be critical for their fitness. So why is it difficult to see the signatures of epistasis in experimental studies of HIV fitness that are largely based on replicative capacity measurements? Can we find new ways to reveal the importance of epistasis for viral evolution? In our manuscript we answer these two questions; we make the following points:

(i) the Potts global sequence covariation model captures a sensitive measure of epistasis: the higher order marginals of the sequence distribution for drug-resistance-associated positions (DRAPS) in HIV proteins exhibit strong effects of epistasis. Furthermore, as we have recently shown, the higher order marginals of evolutionary sequence distributions provide a new and sensitive measure of epistasis for viral proteins and Eukaryotic protein families (see [2]).

(ii) Replicative capacity experiments, in contrast, typically probe the effects of single point mutations in a specific sequence background (laboratory molecular clone) on viral fitness. The epistatic signal from a single point mutation is weak because both the independent and Potts models are constrained to give the same univariate marginals, and because of sequence conservation.

(iii) Double mutant cycle experiments are the classic way to probe epistasis, in principle they quantify the effects of correlation between mutations at two positions. However, in this manuscript we carry out a statistical analysis to show that it is very difficult to quantify epistasis

for HIV proteins using double mutant cycle experiments, because of the small dynamic range of these experiments and consequently the very high precision required to detect epistasis.

Below we address the reviewers' comments in more detail:

<u>Reviewer 1:</u>

In this contribution, the authors use molecular sequence data from four HIV-1 proteins that are important drug targets, to validate a model of the sequence data, and to show the importance of epistasis in predicting physical properties of mutated proteins.
Given the importance of these targets in drug design, and our attempts to understand protein structure and function from sequence data, this is an important area to advance. While some of the findings (importance of epistatic interactions) have been demonstrated for other proteins before, applying this model to HIV sequence data is still necessary. The importance of epistatic effects in the function of the HIV-1 protease has previously been discussed in Gupta & Adami, PLoS Genetics 12(2016) e1005960.

We thank the reviewer for the commentary. We note that we did cite the 2016 Gupta & Adami PLoS Genetics paper in the current manuscript as well as in a previous related paper of ours [3]. In the manuscript under review we focus on the limits to detecting epistasis in viral systems like HIV through experimental measures of fitness and present an alternative approach based on the higher order marginals of the multiple sequence alignment (MSA) distribution, which provides much stronger evidence for epistasis in viruses, and is complementary to an approach based on pairwise mutual information presented in the work by Gupta & Adami. We comment on this in the Conclusion on p. 10-11 of the revised manuscript.

While I find many of the results satisfying, I am somewhat dismayed at the sloppy presentation of model and methods. First of all, the authors refer to the spin model as a Potts model, but it certainly is not that, because the Potts model has interactions only between nearest neighbors. It is also not a standard Ising model (this is what Bialek's group has been calling the model, also Ferguson et al.) but the Ising model is also nearest-neighbor. In the condensed matter literature, this model is the "infinite-range Ising model", because each "spin" can interact with any other spin.

We are glad the reviewer found many of the results presented in our manuscript "satisfying". The reviewer is right that the models initially developed by Potts et al. in the condensed matter physics literature only included nearest neighbor interactions and spin state vectors distributed across a hypersphere [17,18]. In contrast, the “spin” models used in biological physics which correspond to <u>global models of sequence-covariation,</u> are generalizations of the Potts model, for which each "spin" can assume any of 21 possible states (20 amino acid types plus a gap), and each spin can interact with every other spin ("infinite range"). Such spin-models have been well established in the maximum entropy protein sequence-covariation literature and are often referred to simply as the Potts or Ising (in case of just two spins or amino-acid residues, wildtype and mutant) model [3, 5-16]. In accordance with the literature, we refer to this model concisely as simply the Potts model. However, to address the concern of the reviewer, we provide additional commentary about the name on p. 12 of the revised text.

We note that this manuscript does not present any new development of the model but utilizes the model to detect and understand the role of epistasis in shaping the fitness landscape of HIV proteins. The precise spin model and methodology used here is well described in great

detail in previous work from our group [5,15,16], and in the manuscript, we refer the readers to these papers for a more detailed description of the model. To make the manuscript self-sufficient however, we present a brief description of the model and preprocessing steps in the Methods section.

In Eq. (2), the sum does not go to L(L-1)/2, but rather to L. (There should be no uni-code U+2060 inline 398).The authors should make it clear that each site i gets its own alphabet, and what these residues are (at each site) should be listed in a table in the Supplementary.

In Eq. (2), the sum does go to L(L-1)/2 and not L for the couplings, as all possible pairs of sites {i,j} has to be accounted for. Using a double sum (separately for i, and j) would have resulted in the summation going to L, but we had used a single sum to count all possible (${}^{L}C_{2}$) pairs. In the revised manuscript, we use a double sum to make it clear. We thank the reviewer for pointing out the unicode display in line 398, which we have corrected in the revised version. We have also explicitly mentioned that each site i gets its own residue (alphabet), and have provided a link on p. 14 (https://github.com/ComputationalBiophysicsCollaborative/HIV_MSAs) to the sequence MSAs used in this analysis, where the residues are at each site are clearly visible.

On line 510 the authors introduce J^{ij} and h^i, but these are never defined. I imagine these are averages of the h^i(S_k) etc.

We thank the reviewer or pointing this out. The J^{ij} and h^i referred here are actually the couplings, J^ij_SiSj, and fields, h^i_Si, respectively, and not their averages. We have made this clear in the revised text.

On line 512 the authors discuss using a "field-less gauge" due to gauge invariance of the Potts Hamiltonian. That is just nonsense: clearly the authors are using fields h_i that are non-vanishing, just like everybody else.

While what the reviewer says is true for BvH theory (what we call the independent model, see next response), it is not true for the Potts models. For Potts models, there are a set of "gauge transformations" which give us the freedom to set the field values to zero, as long as we make a corresponding change to the pairwise coupling values of the model to compensate, specifically by hia -> 0, Jijab + hia -> Jijab for any choice of j for all b. Please see our presentation of the "fieldless gauge" in Haldane, Levy CPC 2020 [16].

These gauge transformations are very well known in the Potts model literature and have been discussed many times. See for instance, Morcos et al. PNAS 2011 [20] appendix, Ekeberg et al. PRE 2013 [21], Barton et al. Bioinformatics 2016 [8], and in more detail in Cocco et al. Rep. Prog. Phys 2018 [22], section 2.2.3.

Because the "fieldless gauge" is a technical detail of our inference method not explained in full in the manuscript, we have removed it in the revised manuscript to avoid confusion and instead refer to the publication Haldane, Levy CPC 2020 [16] for the full description. The use of the fieldless gauge during inference does not change any of our results, since our results are gauge-invariant.

On line 521, the authors discuss the "univariate marginals h_i", which they don't define. They also very like do not mean h_i, but rather the marginal probabilities p_i from the MSA.

We thank the reviewer for pointing out this typo. We have corrected this in the revised text.

Once you do that, it should occur to you immediately that the mean per-site energy is in fact related to the mean per-site entropy and a term \sum_i \log p_0(i), where p_0(i) is the frequency of the consensus residue. This is the first-order contribution to the free energy. This all follows from Berg-von Hippel (BvH) theory. I realize this sounds cryptic, but I can't give a full elaboration here. It doesn't mean that you don't have to fit all the parameters of the model, but there are additional constraints, which should make the inverse problem a lot easier. Note that in the BvH theory, there is a strong correlation between site energies and the binding affinity of the protein to the sequence. This is no accident. It is for the same reason that there is a correlation between Potts energy and RC of the virus. I'm just stunned that this doesn't appear to be something the literature is aware of.

We thank the reviewer for bringing up these interesting theoretical issues. Although in this short format the reviewer didn't have room to elaborate in detail, we believe the Potts model is more well-developed than the reviewer suggests.

The reviewer refers to Berg-von Hippel (BvH) theory for transcription factor (TF) binding sites, which we are familiar with, particularly with later progress by Michael Lassig and other collaborators. We believe that what the reviewer calls "Berg-von Hippel" theory is functionally identical to what in our manuscript we call the "independent model". For the independent model, there is a field term ("energy level" epsilon_lB in BvH) for each position for each residue, and the probability of a sequence is the exponential of the statistical energy E(S), which is a sum over field terms for that sequence.

We emphasize that we focus instead on the Potts model, which is significantly more complex and powerful than the independent model as it includes pairwise interactions between residues which induce correlations to all orders, i.e. pairwise and higher order correlations. While the two models have many similarities, they also have major differences. For instance, while it is easy to infer the field parameters in BvH simply as the logarithm of the univariate residue frequencies, it well known historically (Cooper, 1990 [28]) that inference for the Potts model (aka inverse Ising inference, graphical models) is NP-hard and requires advanced computational methods. It seems unlikely the reviewer has found an easier exact solution for Potts inference.

Critically, the Potts model allows one to distinguish "direct" from "indirect" epistatic covariation, unlike "mutual information" (MI) scores. The Potts model is best suited to predicting the effects of epistasis in specific backgrounds because it is a "global probability model". See Ref [23] for a discussion of these properties. To briefly summarize, through chains and networks of pairwise interactions the Potts model captures higher-order covariation (triplet, quadruplet, etc.), meaning covariation involving many sites is not simply explainable from the pairwise covariation statistics. Furthermore, we have recently shown that the Potts model can capture the higher order marginals of sequence distributions which provide a new and sensitive measure of epistasis in eukaryotic protein families (see [2]). From "Cluster Expansion" inference methods of obtaining the parameters of a pairwise interaction Potts model, which use the Fano entropy decompositions in Barton et al., 2016 [29], we know that 6th order and higher covariation/entropies are important for modelling protein datasets, and that these are captured

by the pairwise Potts model. The reviewer notes that the BvH sequence energies match binding-affinity well for TFs, but we have previously shown that the BvH/independent model is insufficient to model protein sequence free-energies in which tertiary structure (epistatic residue interaction) is critical, see [14].

Theoretically, the "spin-glass" mathematics of the Potts model are different from that of the BvH model. The reviewer discusses the connections between site-entropies and free-energy in BvH; we refer the reviewer to literature describing such issues for Potts models of proteins, see Refs [24,25]. For example, the coupling factor "lambda" in BvH (1986) [26] is called the "design temperature" in this literature. The Potts model requires a significantly different analysis, however.

Finally, we want to express our appreciation that reviewer #1 had identified himself. We would be glad to continue our discussion with him about different approaches to include correlations in models of biological sequences, but we trust the reviewer understands that, that is not the focus of our manuscript.

When showing site-entropy (as in Figs. S2A and B) it should be specified where this uses the reduced alphabet or not.

A reduced alphabet is used to calculate the site-entropies in Figure S2A and B, which we have specified in the revised supplement.

I have no reason to suspect that the authors used the model incorrectly, nor do I doubt their results. But the sloppiness in the model description needs to be fixed.

We appreciate the confidence the reviewer has expressed that our use of the Potts model and the results we present are correct. We thank the reviewer for finding some typos, but we think the statement that there was "sloppiness in the model description" is unwarranted. In the revised manuscript, we have included additional information about the origin of the name "Potts model" as it is used in biological physics and corrected the typos pointed out by the reviewer.

<u>Reviewer 2:</u>

In this highly technical work Levy and coworkers assess the role of "epistasis" in Potts model of clinically derived HIV sequences including drug resistant variants. They explore various measures from purely sequence-based such as distribution of hamming distances or frequencies of “sequence marginals" (i.e. NON-CONTIGUOUS sub sequences of various lengths) to various measures of experimentally determined fitness such as replicative capacity etc.

The reviewer comments that our manuscript is "highly technical". We have made revisions to the introduction and conclusion sections in the manuscript in an effort to make the story more accessible and to explain more clearly why the results and conclusions are important. The primary thrust of the manuscript is to show that, because of sequence conservation, it is very difficult to detect epistasis in viruses like HIV using conventional experimental measures of fitness, particularly replicative capacities which typically involve only single point mutations on a specific molecular clone that is very close to the consensus sequence. We also use a simple statistical model to demonstrate why it is difficult to detect epistasis in HIV using double mutant

cycles due to the small dynamic range of these experiments and consequently the high precision that is required to detect the effects of epistasis. To emphasize this point, we have revised the title. We also present a metric based on higher order marginals of the MSA, which provides strong evidence for epistasis in HIV. For a more complete description of this new metric see McGee et al., 2020 [2].

For all these quantities the authors compare the performance of independent site model (where only "external fields" are included in the Potts Hamiltonian to full "epistatic" model where pairwise couplings are also included. It is important to note that pairwise coupling model includes much more sequence information than independent interaction model so that comparisons with sequences on which the model has been trained in the first place should include this factor.

There appears to be some misunderstanding. First, the independent model and the Potts pairwise interaction model, do not in general share the same field terms. The independent model is parameterized to reproduce the univariate marginals of the MSA, while the Potts model is parameterized to reproduce the bivariate marginals, and therefore it also reproduces the univariate marginals. If the coupling terms are removed from the pairwise Potts model, the remaining fields-only expression does not reproduce the univariate marginals, except in a special choice of gauge. Indeed, the pairwise Potts model contains more parameters than the independent model. However, the important new result reported in our manuscript is that the Potts pairwise model is able to predict the higher order marginals (beyond pairwise) of HIV protein sequence MSAs, up to the detection limit imposed by finite sampling. The pairwise model is <u>not</u> parameterized to reproduce these higher marginals. We find that the pairwise interaction Potts model is both necessary and sufficient to capture the higher order correlations inherent in the HIV sequence datasets that are the focus of this manuscript. For an extensive discussion of this important result in a more general context, see Ref [14].

They used Newton optimization to as developed in Ref 31 to determine parameters of the Potts Hamiltonian to find best fit between generated sequences and the data in terms of 1- and 2- site correlations.

While our solution to the inverse inference problem is based on the methodology first described in Ref 31 (in the original manuscript), several changes and improvements have been made in order to implement the method on GPUs as described in Ref [16] (*Haldane et al.,* 2021). Using GPUs to solve the inverse inference equations by Monte-Carlo methods which make fewer approximations, leads to solutions that are more accurate and robust.

While the work involves considerable amount of calculations it is inconclusive and lacks message. Fig.3 shows that epistatic Potts predicts sequence marginals better than independent for some proteins but not the others. What is the meaning of this finding? Given that analysis includes non-contiguous marginals is it surprising that including more information from the data gives you better prediction of the data itself? Is this factor included in the statistical analysis? What does the ability to predict input data tell us generally about the capacity of the Potts model to predict important experimentally measurable quantities?

We regret that we have apparently not presented our work and conclusions clearly enough and have revised the manuscript in an effort to improve the presentation. The reviewer's comment touches on two issues. The first concerns the ability of the pairwise Potts model to correctly predict "the input data itself", and the second concerns the ability of sequence-based models

like the Potts model to predict experimental quantities like replicative capacity. The pairwise Potts model is parameterized to reproduce the bivariate marginals of the target protein MSA distribution. The fact that it can reproduce high order marginals is certainly not guaranteed and is surprising to many people working in the field. Furthermore, there is no evidence to suggest that a sequence co-variation model which includes triplet terms can reproduce sequence and sub-sequence probabilities any better than a pairwise interaction Potts model. In other words, the pairwise Potts interaction model is both necessary and sufficient to reproduce sequence probabilities as well as can be determined in the presence of finite sampling limitations [2, 14]. Concerning the second issue, the relationship between the Potts model and experimentally measurable quantities, it is a fundamental assumption that the prevalence of a sequence in the population is a measure of its fitness, and the claim is that the Potts model provides the best estimate of sequence prevalences currently available. Because of the vast size of sequence space it is not possible to directly "measure" the prevalence of most sequences, we use the ability of the Potts model to capture the prevalence of higher order marginals up to the finite sampling limit as a proxy for the prevalence of complete sequences. We therefore expect the Potts model to be able to predict measurable quantities which track fitness. We have explained why replicative capacity measurements based on single point mutations are not well suited to distinguish an epistatic model from an independent model.

On that front subsequent analysis is even less conclusive. Clearly Potts Hamiltonian (epistatic and independent alike) does fairly poor job in predicting experimental measures of "fitness" as defined in various experimental approaches. Does it suggest that epistasis does not play a role in sculpting fitness landscape (unlikely) or that pairwise Potts with spin-like Hamiltonian is inadequate to predict experimentally fitness? Authors argument that MSA reflects on other measure of fitness than observed in lab experiments while potentially plausible does not generate specific insights as to how-to interpret Potts Hamiltonian results and/or predictions and what kind of experiments should be designed to support or falsify those.

First, only the pairwise model is referred to as the Potts model in accordance with the current literature, and the model devoid of pairwise coupling terms is called the "independent" model. Secondly, we disagree with the reviewer's comment that the Potts Hamiltonian model does a fairly poor job in predicting experimental measures of fitness. Barring one particular experimental result where the experimental data appears to be problematic (as shown in the supplementary material that they do not correlate with other experimental results for the same mutations), the Potts model predicts experimental replicative capacities relatively well with an average correlation coefficient ~ 0.6 We have now removed the problematic experimental dataset [4] from our analysis to make the message clear, but we do retain the comment about this dataset in the supplementary material. It is also to be noted that our MCMC implementation of the Potts model on GPUs makes use of few analytical assumptions and predicts experimental data with a higher correlation coefficient than other (inferred using different methodologies) Potts models in the literature for the same dataset [27]. It is not that the Potts model is inadequate to predict experimental fitness as the reviewer suggests, but the fact that the independent model performs almost equally well in capturing experimental fitness measures for viral proteins, which has also been observed in the highly cited work by Riesselman et al. [1]. In contrast to many different kinds of fitness experiments performed on eukaryotic protein families, where the Potts model is superior to the independent model, our work shows that for viral proteins, replicative capacity measurements do not distinguish between correlated and independent fitness models because they primarily involve only single point mutations and because high evolutionary conservation within a single viral protein family (as compared with between eukaryotic protein families) masks the effects of epistasis.

My overall assessment that this work, while reporting some interesting and extensive calculations does not offer relevant biological insight.

We think that the reviewer misunderstood major portions of the manuscript and have tried through revision to make our story and conclusions clearer.

References:

1. Riesselman AJ, Ingraham JB, Marks DS. Deep generative models of genetic variation capture the effects of mutations. Nature methods. 2018;15(10):816–822.
2. McGee, F., Novinger, Q., Levy, R. M., Carnevale, V., & Haldane, A. (2020). Generative Capacity of Probabilistic Protein Sequence Models. *arXiv preprint arXiv:2012.02296*. Accepted, Nature Communications.
3. Flynn WF, Haldane A, Torbett BE, Levy RM. Inference of epistatic effects leading to entrenchment and drug resistance in hiv-1 protease. Molecular biology and evolution. 2017;34(6):1291–1306.
4. Al-Mawsawi LQ, Wu NC, Olson CA, Shi VC, Qi H, Zheng X, et al. High-throughput profiling of point mutations across the HIV-1 genome. Retrovirology. 2014;11(1):124.
5. Haldane A, Flynn WF, He P, Vijayan R, Levy RM. Structural propensities of kinase family proteins from a Potts model of residue co-variation. Protein Science. 2016;25(8):1378–1384.
6. Shekhar K, Ruberman CF, Ferguson AL, Barton JP, Kardar M, Chakraborty AK. Spin models inferred from patient-derived viral sequence data faithfully describe HIV fitness landscapes. Physical review E. 2013;88(6):062705.
7. Mann JK, Barton JP, Ferguson AL, Omarjee S, Walker BD, Chakraborty A, et al. The fitness landscape of HIV-1 gag: advanced modeling approaches and validation of model predictions by in vitro testing. PLoS computational biology. 2014;10(8):e1003776.
8. Barton JP, De Leonardis E, Coucke A, Cocco S. ACE: adaptive cluster expansion for maximum entropy graphical model inference. Bioinformatics. 2016;32(20):3089–3097.
9. Louie RH, Kaczorowski KJ, Barton JP, Chakraborty AK, McKay MR. Fitness landscape of the human immunodeficiency virus envelope protein that is targeted by antibodies. Proceedings of the National Academy of Sciences. 2018;115(4):E564–E573.
10. Barton JP, Kardar M, Chakraborty AK. Scaling laws describe memories of host–pathogen riposte in the HIV population. Proceedings of the National Academy of Sciences. 2015;112(7):1965–1970.
11. Barton JP, Goonetilleke N, Butler TC, Walker BD, McMichael AJ, Chakraborty AK. Relative rate and location of intra-host HIV evolution to evade cellular immunity are predictable. Nature communications. 2016;7:11660.
12. Biswas A, Haldane A, Arnold E, Levy RM. Epistasis and entrenchment of drug resistance in HIV-1 subtype B. eLife. 2019;8.
13. Butler TC, Barton JP, Kardar M, Chakraborty AK. Identification of drug resistance mutations in HIV from constraints on natural evolution. Physical Review E. 2016;93(2):022412.
14. Haldane A, Flynn WF, He P, Levy RM. Coevolutionary landscape of kinase family proteins: sequence probabilities and functional motifs. Biophysical journal. 2018;114(1):21–31.
15. Haldane A, Levy RM. Influence of multiple-sequence-alignment depth on Potts statistical models of protein covariation. Physical Review E. 2019;99(3):032405.
16. Haldane A, Levy RM. Mi3-GPU: MCMC-based inverse ising inference on GPUs for protein covariation analysis. Computer Physics Communications. 2020; p. 107312.
17. Potts RB, Ward JC. The combinatrial method and the two-dimensional Ising model. Progress of Theoretical Physics. 1955;13(1):38–46.
18. Wu FY. The potts model. Reviews of modern physics. 1982;54(1):235.

19. Berlin TH, Kac M. The spherical model of a ferromagnet. Physical Review. 1952;86(6):821.
20. Morcos F, Pagnani A, Lunt B, Bertolino A, Marks DS, Sander C, et al. Direct-coupling analysis of residue coevolution captures native contacts across many protein families. Proceedings of the National Academy of Sciences. 2011;108(49):E1293–E1301.
21. Ekeberg M, Lo ̈vkvist C, Lan Y, Weigt M, Aurell E. Improved contact prediction in proteins: using pseudolikelihoods to infer Potts models. Physical Review E. 2013;87(1):012707.
22. Cocco S, Feinauer C, Figliuzzi M, Monasson R, Weigt M. Inverse statistical physics of protein sequences: a key issues review. Reports on Progress in Physics. 2018;81(3):032601.
23. Weigt M, White RA, Szurmant H, Hoch JA, Hwa T. Identification of direct residue contacts in protein–protein interaction by message passing. Proceedings of the National Academy of Sciences. 2009;106(1):67–72.
24. Pande, V. S., Grosberg, A. Y., & Tanaka, T. (1997). Statistical mechanics of simple models of protein folding and design. *Biophysical journal*, *73*(6), 3192-3210.
25. Pande, V. S., Grosberg, A. Y., & Tanaka, T. (2000). Heteropolymer freezing and design: towards physical models of protein folding. *Reviews of Modern Physics*, *72*(1), 259.
26. Von Hippel, P. H., & Berg, O. G. (1986). On the specificity of DNA-protein interactions. *Proceedings of the National Academy of Sciences*, *83*(6), 1608-1612.
27. Zhang Th, Dai L, Barton JP, Du Y, Tan Y, Pang W, et al. Predominance of positive epistasis among drug resistance-associated mutations in HIV-1 protease. PLoS genetics. 2020;16(10):e1009009.
28. Cooper, G. F. (1990). The computational complexity of probabilistic inference using Bayesian belief networks. *Artificial intelligence*, *42*(2-3), 393-405.
29. Barton, J. P., De Leonardis, E., Coucke, A., & Cocco, S. (2016). ACE: adaptive cluster expansion for maximum entropy graphical model inference. *Bioinformatics*, *32*(20), 3089-3097.
