# Supplementary material for: Limits to detecting epistasis in the fitness landscape of HIV
Source: PLoS One. 2022 Jan 18;17(1):e0262314. doi: 10.1371/journal.pone.0262314 (PMC8765623; doi:10.1371/journal.pone.0262314)
Supplement: Attachment — Submitted filename: PLOS_One_Response_to_Reviews.pdf [file pone.0262314.s004.pdf]

PONE-D-21-33103: Limits to detecting epistasis in the fitness landscape of HIV
Authors: Avik Biswas, Allan Haldane, Ronald M levy

Dated: 12/17/2021

Response to Reviews:

The work of Biswas et al. focuses on the use of data-driven models of protein sequence composition to characterize the fitness landscape of proteins, particularly HIV proteins that are commonly targeted by antiretroviral drugs. This work deals with a common and relevant problem in the field of amino acid coevolution where both sequence data (or its diversity) is scarce, and where experimental measures of fitness are limited by experimental constraints. This extensive study has two important results: 1) higher order marginals can be recovered with a pairwise model and that such higher order marginals indeed play a role in the fitness landscape of viral proteins. 2)It sheds light on why existent experimental data trying to capture epistatic effects of mutations is hard to model due to the limited dynamic range of these experiments.

Being this a revision of an initial submission, I was able to review previous reviewers comments and the accompanying responses and changes. My assessment is that the responses to the reviewer comments were appropriate and the changes did in fact improve the clarify of the presentation. My general assessment from the previous reviewers was that they did not have enough background in the recent developments in the field of amino acid coevolution and were focused on challenging the model development itself which has been already established and refined in the literature. This revision is technically sound but also does a good job presenting the biological implications of their results. In conclusion, I think this study is relevant for the field and explains several open questions concerning the application of sequence Potts models to viral proteins. I find this study relevant and of use, especially in these times where understanding the fitness landscapes of proteins related to infectious diseases in a matter of pressing public health.

Given the extensive changes toward this revision, I only have a few questions and comments that if responded could make this manuscript more clear.

We thank the reviewer for the thoughtful commentary and for highlighting the main points of the manuscript. The reviewer finds the study "relevant and of use". Below we provide a point-by-point response to the questions and comments raised by the reviewer. We have also updated the manuscript with changes made in response to the current reviewer comments highlighted in "cyan", while changes made to the previous reviewers' comments are highlighted in "yellow".

General:

1. The finding that the Capsid shows a higher correlation with the Potts model is interesting. A similar correlation has been observed for Capsids on other types of viruses (AAV) providing further evidence of the relevance of Potts models in these types of studies. I suggest citing this additional study too (https://doi.org/10.1016/j.bpj.2020.12.018).

We thank the reviewer for the suggestion and have added the citation in the updated manuscript.

2. Data Processing. It is stated that positions with more than 1% gaps were removed. This seems to me like a very stringent cutoff, can the authors provide an explanation of why more than1% is too much?

We had not used the correct wording, "gap"; while referring to the "dot" character in HIV protein sequence alignments available from the Stanford HIV drug resistance database, which represents an unsequenced position in the sequence. Such positions (columns in the multiple sequence alignment) with more than 1% "dots" or missing amino characters were removed from the MSA, so that the subsequent Potts model built on the MSA would not have spurious correlations between missing amino acids/unsequenced positions and amino acid residues at other positions. Similarly remaining sequences (rows in the MSA) with "dots" (a small fraction of the MSA) after the first filtration step, were then removed to preserve the quality of sequences. We have made this clear by revising the previous statement in the Materials and Methods section:
" MSA columns with more than 1% ``dots" ('.') which represent unsequenced positions in the sequences are removed to avoid spurious correlations in the subsequent Potts model built on the MSA. Remaining sequences with any "dots" or unsequenced positions are then removed."

3. Data processing. The statement "Sequences with insertions and deletions are removed" is not clear to me. Do you mean the positions with insertions and deletions? Or are you removing any complete sequence that has an insertion or deletion? Somehow this does not makes sense to me.

Complete sequences with insertions or deletions are removed. There are only few such sequences and removing them doesn't affect the multiple sequence alignment (MSA) statistics much; for example, less than 1% (~0.3%) of the sequences in the Reverse Transcriptase MSA contain insertions or deletions. On the other hand, keeping these sequences in the MSA would have complicated the subsequent model building, without improving the statistics. We have now made this clear in the revised text:
" Complete sequences with any insertions ('#') or deletions ('~') are removed. Such sequences form a small fraction (<1%) of the MSA and removing them doesn't significantly affect the MSA statistics. "

4. Alphabet reduction. I wonder if Equation 5 could be improved by including an index instead of the explicit realizations like Q=20, my understanding is that after each iterative step, Q will be reduced with respect to the previous step right? If this is true then it should be noted that this is not only valid for a change from 21 to 20. If I misinterpreted it, then it is possible that the equation needs some further clarification.

The reviewer is right. In each iterative step, the alphabet is reduced by 1 from a Q-letter alphabet in the previous step to a Q-1 letter alphabet. To make this clear, we have modified Equation 5 with an index instead of explicit realizations as below:

$$MI_{RMSD} = \sqrt{\frac{1}{N}\sum_{ij}\left(MI_{Q}^{ij} - MI_{Q-1}^{ij}\right)^{2}}$$

5.Alphabet reduction. The authors compared the model with alphabet reduction with the model using full parameters. If the model with the full parameters was inferred, then what was the motivation to reduce the alphabet?

We apologize for the confusion. We only compared the Mutual Information (MI) of the MSA encoded in the full 21-letter (20 amino acids + 1 gap character) alphabet to the MI of the MSA in a reduced alphabet. The Potts model is only inferred based on the MSA in the reduced alphabet for computational efficiency

and not inferred based on the MSA encoded in the full 21-letter alphabet. In the most recent work in our group, we are using the full alphabet. The project described in this manuscript was carried out using a reduced alphabet. We do not believe the alphabet reduction affects any of the results presented in this manuscript.

6. In the section statistical robustness of HIV Potts models, the concept of Signal-to-noise ratio is introduced and scores for the different protein systems are presented. Although it is mentioned that SNR depends on several factors, it would be good to have a more concrete definition or point out to previous work where it is defined.

We have now included a reference to previous work from the group which gives a more elaborate definition of the SNR and the several factors it depends on:
" The SNR for Potts models fit to protein sequences is discussed in more detail in [54]. "

Minor:

1*. The use of the term favorability/ disfavorability is a bit awkward, I would consider another term.*

We have used the terms "favorability/disfavorability" for a mutation in its specific sequence background, in keeping with previous published work (*Biswas et al*., eLife, 2019). The Potts model describes the prevalence landscapes of the protein sequences, and the model ΔEs best describe the favorability/disfavorability of mutations in a given sequence background. Alternatively, using terms like stabilizing/destabilizing (*Flynn et al*., MBE, 2017) can lead to further confusion as they can be interpreted to be pertaining specifically to protein stabilities.

2. *Conventions, should "Fig 1" be spelled "Fig. 1" ?*

We have now renamed all figures in the manuscript according to the convention, for example Fig 1 as Fig. 1.

3. *Page 5, line 149. Change ".. higher entropies in Supplementary File 1 Fig 2 A" to ".. higher entropies (Supplementary File 1 Fig 2 A)."*

We have modified the sentence in the Main text.

4. *Page 5, line 157. Change "This is suggestive that strong couplings .." to "This is suggestive of strong couplings"*

We thank the reviewer for pointing out the typo and have corrected it in the revised text.

5. *Page 11, line 364. correct "changes in CA perhaps has a .." to "changes in CA perhaps have a .."*

We have corrected the typo in the revised text.

6. *NRTIs is only defined in a figure caption, I suggest to define it also in the main text.*

We thank the reviewer for pointing this out and have now defined all the major drug-classes used in antiretroviral therapy including NRTIs in the Main text lines 432-436.

7. *Data processing. The concept of "deletes" is used instead of "deletion", I would simply use deletions*.

We have changed the word "deletes" to "deletions".

8. *Page 13, line 444. Add a period after "filtered out."*

Added a period.

9. *Page 13, correct "drug resistance mutations is not yet" with "drug resistance mutations are not yet"*

Corrected.

10. *Mutation information section. Replace "including list of .." with " including a list of .."*

We have modified the main text replacing "including list of .." with "including a list of .." as pointed out by the reviewer.

11. *Subsection title. Use lower case across titles, e.g. Change "Statistical Robustness of HIV" with "Statistical robustness of HIV .."*

We have now modified the font for the subsection titles accordingly.

12. *Change title in the SI to be compatible with the manuscript.*

We have now changed the title of the SI to be compatible with the manuscript.

13. *What is the need of having two distinct Supplementary files? It seems to me that the two files can be combined into a single document.*

The supplementary files are kept separate for ease of readability. Supplementary file 1 contains the details of many methods, supplementary data, and figures. Supplementary file 2 on the other hand contains only supplementary tables and figures pertaining to double mutant cycles showing that most strongly coupled pairs of mutations predicted by the Potts model contain drug-resistance associated mutations studied in the literature.
Note that there was a slight error in Figure S2A in Supplementary File 2, in marking the standard deviations, which we have now corrected and modified in the revised version. This does not affect any of the results.
